# Supplementary material for: Fecal Microbiota and Diet Composition of Buryatian Horses Grazing Warm- and Cold-Season Grass Pastures
Source: Microorganisms. 2023 Jul 30;11(8):1947. doi: 10.3390/microorganisms11081947 (PMC10459317; doi:10.3390/microorganisms11081947)
Supplement: Supplementary file 1 [file microorganisms-11-01947-s001.zip › Figure S1.pdf]

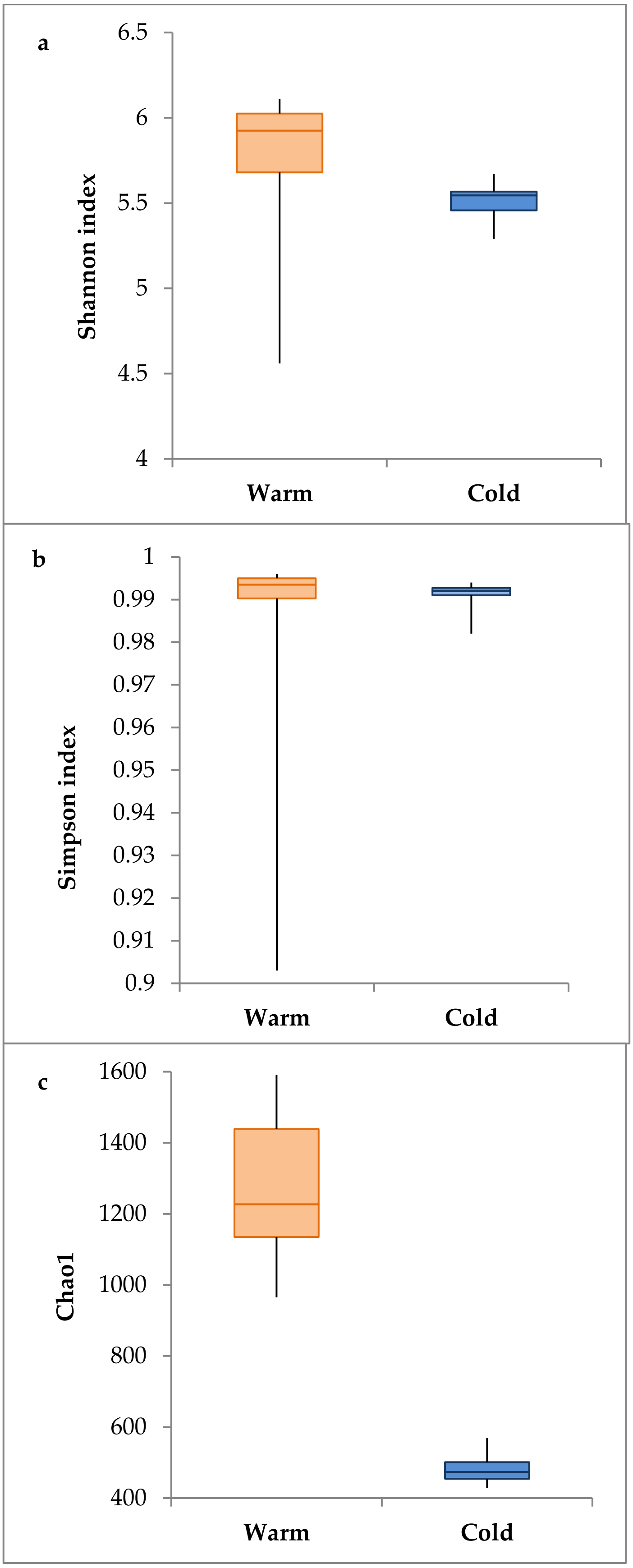

Figure S1. Boxplots of differences in alpha diversity between the warm- and cold season. All data are presented as the mean ± standard error. (a) Shannon diversity index; (b) Simpson diversity index; (c) Chao1.
