# Supplementary material for: Fecal Microbiota and Diet Composition of Buryatian Horses Grazing Warm- and Cold-Season Grass Pastures
Source: Microorganisms. 2023 Jul 30;11(8):1947. doi: 10.3390/microorganisms11081947 (PMC10459317; doi:10.3390/microorganisms11081947)
Supplement: Supplementary file 1 [file microorganisms-11-01947-s001.zip › Table S1.pdf]

Table S1. Statistical reads characteristics

Sample	Sample_ID	Raw sequences (read pairs)	Merged read pairs	Filtered 16S rRNA gene sequences
S1	151-22	38192	32185	26305
S2	154-22	8586	7246	5898
S3	155-22	10611	8953	7374
S4	156-22	9859	8414	6817
S5	158-22	12697	10810	9053
S6	159-22	5337	4532	3728
S7	160-22	9752	8542	7211
S8	161-22	8686	7393	5884
S9	162-22	9571	8269	6751
S10	163-22	6975	5900	4859
S11	164-22	9818	8359	6753
S12	165-22	9598	8122	6541
S13	166-22	18271	15893	12997
S14	167-22	4518	3864	3192
W1	horse.metagenome.V4.25.R1	14245	11671	10587
W2	horse.metagenome.V4.26.R1	12786	10325	9781
W3	horse.metagenome.V4.27.R1	15694	12978	11983
W4	horse.metagenome.V4.28.R1	16477	12906	11816
W5	horse.metagenome.V4.29.R1	14723	12332	11416
W6	horse.metagenome.V4.30.R1	15286	12231	11414
W7	horse.metagenome.V4.31.R1	15075	12302	11236
W8	horse.metagenome.V4.32.R1	13772	11286	10218
W9	horse.metagenome.V4.33.R1	13898	11358	10453
W10	horse.metagenome.V4.34.R1	15049	12162	11141
W11	horse.metagenome.V4.35.R1	14959	12449	11534
W12	horse.metagenome.V4.36.R1	15636	13001	11988
W13	horse.metagenome.V4.37.R1	14702	11943	10916
W14	horse.metagenome.V4.38.R1	15965	12973	11618
