# Supplementary material for: Fecal Microbiota and Diet Composition of Buryatian Horses Grazing Warm- and Cold-Season Grass Pastures
Source: Microorganisms. 2023 Jul 30;11(8):1947. doi: 10.3390/microorganisms11081947 (PMC10459317; doi:10.3390/microorganisms11081947)
Supplement: Supplementary file 1 [file microorganisms-11-01947-s001.zip › Table S2.pdf]

Table S2.Comparison of mean relative abundance at phylum level between cold- and warm-season.

Taxon	Cold-season	Warm-season	P-value
Firmicutes	62.95	50.74	<0.001*
Bacteroidota	25.33	27.90	0.092
Actinobacteriota	2.61	1.45	0.027
Campylobacterota	2.48	0.14	<0.001*
Desulfobacterota	2.23	0.21	<0.001*
Spirochaetota	2.23	2.20	0.056
Proteobacteria	1.25	2.88	0.123
Deferribacterota	0.33	0	0.003*
Verrucomicrobiota	0.21	7.00	<0.001*
Euryarchaeota	0.08	0.65	0.001*
Fibrobacterota	0.02	1.34	0.002*
Cyanobacteria	0.02	2.86	<0.001*
Halobacterota	0	1.550	0.006
Synergistota	0	0.38	0.002*

*Level of significance was P≤0.003 after Bonferroni correction for multiple comparisons
