# Supplementary material for: Fecal Microbiota and Diet Composition of Buryatian Horses Grazing Warm- and Cold-Season Grass Pastures
Source: Microorganisms. 2023 Jul 30;11(8):1947. doi: 10.3390/microorganisms11081947 (PMC10459317; doi:10.3390/microorganisms11081947)
Supplement: Supplementary file 1 [file microorganisms-11-01947-s001.zip › Table S3.pdf]

Table S3.Comparison of mean relative abundance at family level between cold- and warm-season.

Taxon	Cold-season	Warm-season	P-value
[Eubacterium] coprostanoligenes group	2.77	2.61	0.334
Acidaminococcaceae	0.34	1.94	<0.001*
Akkermansiaceae	0.11	0.37	0.008
Anaerovoracaceae	0.63	1.83	<0.001*
Bacteroidaceae	1.61	0.12	0.0002*
Bacteroidales RF16 group	0.12	1.26	<0.001*
Butyricicoccaceae	0.84	0.09	0.0006
Christensenellaceae	1.50	2.32	0.064
Desulfovibrionaceae	2.22	0.17	<0.001*
Eggerthellaceae	1.13	0.95	0.193
Erysipelatoclostridiaceae	3.15	1.17	0.011
Erysipelotrichaceae	4.24	0.96	<0.001*
F082	0.05	3.54	<0.001*
Fibrobacteraceae	0.02	1.34	0.0007*
Lachnospiraceae	11.44	13.11	0.111
Lactobacillaceae	11.47	1.86	<0.001*
Methanobacteriaceae	0.08	0.65	0.0002*
Muribaculaceae	6.43	0.14	<0.001*
Order_Clostridia UCG-014	3.45	0.65	<0.001*
Order_Gastranaerophilales	0.02	2.86	<0.001*
Order_RF39	0.74	0.19	<0.001*
Oscillospiraceae	7.75	9.56	0.078
Peptostreptococcaceae	1.77	0.01	<0.001*
Prevotellaceae	10.91	6.72	<0.001*
Rikenellaceae	4.52	9.11	0.0003*
Ruminococcaceae	9.43	3.18	<0.001*
Saccharimonadaceae	0.04	0.16	0.0003*
Selenomonadaceae	0.77	0.22	<0.001*
Spirochaetaceae	2.21	2.12	0.431
UCG-010	0.06	3.89	<0.001*

*Level of significance was P≤0.003 after Bonferroni correction for multiple comparisons
