# Supplementary material for: Fecal Microbiota and Diet Composition of Buryatian Horses Grazing Warm- and Cold-Season Grass Pastures
Source: Microorganisms. 2023 Jul 30;11(8):1947. doi: 10.3390/microorganisms11081947 (PMC10459317; doi:10.3390/microorganisms11081947)
Supplement: Supplementary file 1 [file microorganisms-11-01947-s001.zip › Table S4.pdf]

Table S4.Comparison of mean relative abundance at genus level between cold- and warm-season.

Taxon	Cold-season	Warm-season	P-value
[Eubacterium]_coprostanoligenes_group	2.19	2.71	0.635
*Christensenellaceae*_R-7_group	1.50	2.29	0.002*
*Clostridia*_UCG-014	3.45	0.63	<0.001*
Lachnoclostridium	0.61	0.60	0.928
*Lachnospiraceae*_AC2044_group	0.42	0.77	<0.001*
*Lachnospiraceae*_NK4A136_group	1.25	1.92	0.014
Ligilactobacillus	3.54	1.07	<0.001*
NK4A214_group	1.19	4.56	<0.001*
Prevotella	1.14	1.07	0.889
*Rikenellaceae*_RC9_*gut_group*	3.91	7.77	<0.001*
Ruminococcus	3.19	1.11	<0.001*
Treponema	2.04	2.05	0.982
UCG-002	0.60	2.14	<0.001*
UCG-005	2.20	2.03	0.754

*Level of significance was P≤0.003 after Bonferroni correction for multiple comparisons
