# Supplementary figures and images for: Fecal Microbiota and Diet Composition of Buryatian Horses Grazing Warm- and Cold-Season Grass Pastures
Source: Microorganisms. 2023 Jul 30;11(8):1947. doi: 10.3390/microorganisms11081947 (PMC10459317; doi:10.3390/microorganisms11081947)

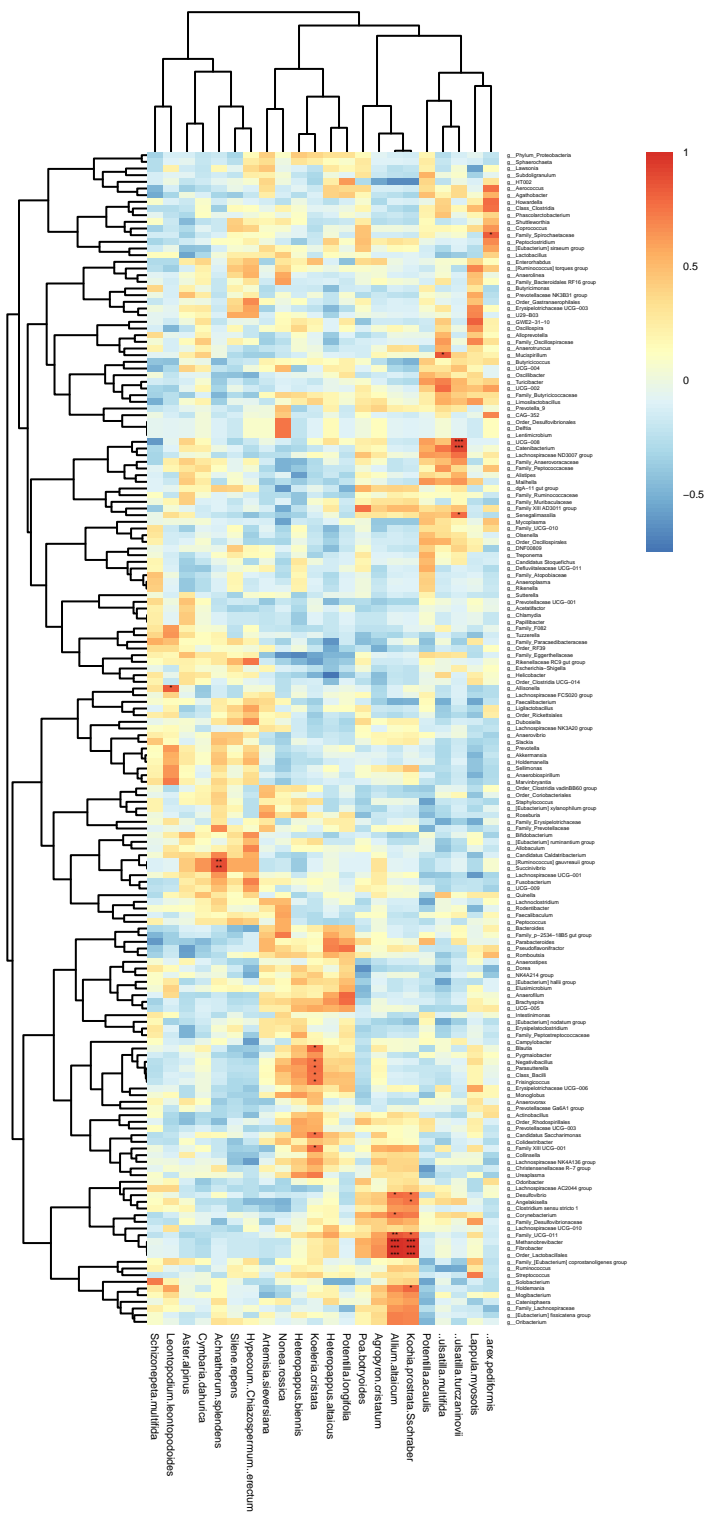

Supplement: Supplementary file 1 [file microorganisms-11-01947-s001.zip › Figure S3.pdf]
